# From prescription to practice: improving patient access and adherence to nature-based clinical interventions

**DOI:** 10.3389/fpubh.2026.1749341

**Published:** 2026-02-25

**Authors:** Max Heimlich-McQuarters, Sarayu Chandra Mouli, Coral Lozada, Weichuan Dong, Jay E. Maddock, Sadeer Al-Kindi

**Affiliations:** 1 Center for Health & Nature, Houston, TX, United States; 2 Department of Cardiology, Houston Methodist, Houston, TX, United States; 3 School of Public Health, Texas A&M University, College Station, TX, United States

**Keywords:** access, clinical practice, community health, greenspaces, health disparities, nature prescriptions

## Abstract

**Background:**

Rooted in historical healing traditions and backed by growing clinical evidence, nature-based prescriptions (GRx) are gaining recognition as low-cost, multifaceted strategies to improve disease prevention. These prescriptions encourage healthcare practitioners to harness the curative properties of nature through structured regimes. However, while many countries have piloted successful GRx programs, widespread integration into clinical practice remains limited, especially in vulnerable communities.

**Objective:**

We conducted a narrative review synthesizing existing evidence to examine the gap between clinician-initiated GRx and practice. We particularly focus on healthcare system integration, adherence, and equity considerations.

**Methods:**

Sources were identified using academic databases including Google Scholar, Scopus and PubMed alongside gray literature such as government documents and private programs. Evidence was synthesized narratively to identify recurring implementation-relevant patterns across clinical, environmental, and patient-level contexts.

**Results:**

Our review found that despite the growing adoption of GRx, their integration into routine clinical practice remains largely stagnant. This has largely been due to recurring patterns related to structured frameworks, limited clinical infrastructure, and inequitable access to greenspace. Across all settings, patient follow-through is consistently hindered due to transportation, safety and time constraints, further exacerbated by environmental and socioeconomic barriers. Based on recurring patterns identified in literature, we propose a practice-informed call-to-action framework grounded in three pillars: access to resources, value and return on investment, and accountability.

**Conclusion:**

By expanding resource access, embedding GRx into clinical workflows, building cross-sector partnerships and strengthening accountability mechanisms, GRx can move beyond its niche status to become a scalable, equitable, and standardized part of preventive care.

## 1 Introduction

Nature has long been used as a healing modality, with roots dating back to Ancient Greek healing gardens that supported physical and spiritual wellbeing (1). This practice continued in 18th century Germany with *Kur*, or therapeutic bathing (2). Today, healing gardens play a transformative role in restoration, recovery and stress relief for patients and staff alike. For instance, specialized cancer gardens provide patients undergoing chemotherapy with soothing environments for stress relief (3). Beyond clinical care, growing research supports daily exposure to nature through lifestyle interventions that improve metabolic, physical, and mental outcomes (4, 5). Rapid urbanization, changing lifestyles, and environmental stressors have reduced access to high-quality greenspace and natural healing environments (6, 7). Urban areas prioritize buildings over nature, while rural areas, though rich in greenspace, lack access, therapeutic design, and safety infrastructure. These disparities contribute to nature deficits and worsen overall health and longevity across both populations (7, 8). In response, healthcare professionals are increasingly acknowledging the value of nature in preventive care (9). With over 25% of adults worldwide not meeting recommended physical activity levels (10, 11), outdoor environments such as walkable green neighborhoods, trails, and parks are often described as supportive of sustained and enjoyable physical activity (12). This has led to the rise of nature-based prescriptions (GRx), which recommends patients to engage in activities like forest walks, outdoor activity or gardening as supplements to traditional allopathic medicine (13). From the National Health Service Shetland Program in Scotland to *Shinrin-yoku* in Japan, GRx initiatives have been successfully implemented in various parts of the world (14, 15). As healthcare systems seek cost-effective, low-risk interventions, GRx offer potential benefits for prevention, health equity, and population well-being.

However, evidence suggests that the potential of GRx is often constrained by real-life conditions. Many patients, especially those from marginalized communities, face persistent challenges to participate in prescribed nature-based activities due to limited access to safe greenspaces, transportation challenges, environmental hazards, and neighborhood safety concerns (16). Even these barriers are not uniformly experienced. LGBTQ+ individuals, members of the BIPOC communities, older adults, and other racialized communities may face worser social-norm constraints when accessing public greenspaces (17). While enthusiasm for GRx continues to grow, less is known about how these programs are integrated into healthcare systems, or why participation remains inconsistent despite clinical endorsement (8, 18, 19).

## 2 Significance and objectives

**Figure 1:**
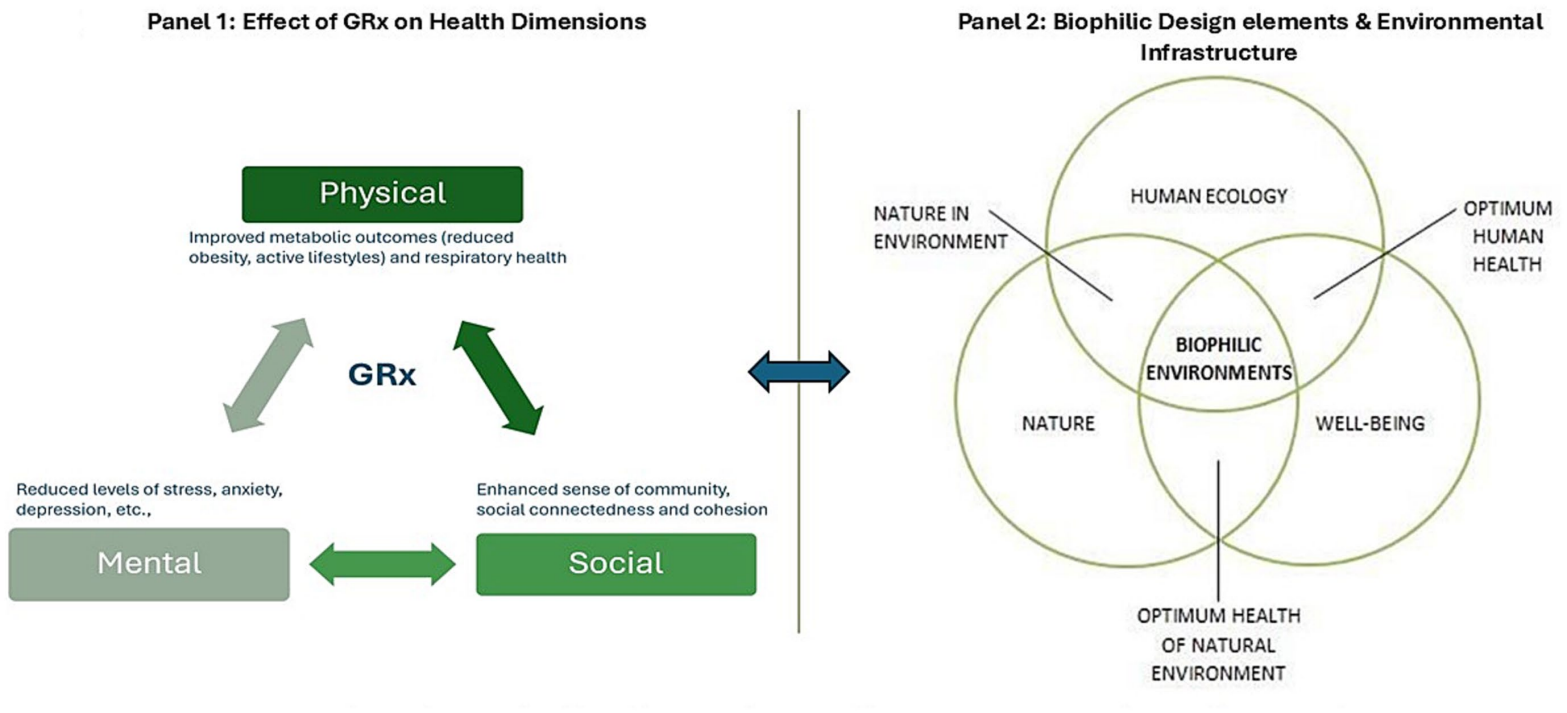
Dimensions of biophilic designs and GRx. Conceptual framework illustrates how biophilic design and environmental infrastructure create enabling conditions for routine nature exposure, which support the feasibility and effectiveness of Green Prescriptions (GRx). GRx functions as a translational mechanism linking designed environments to physical, mental, and social health outcomes, with outcomes feeding back into clinical practice and planning investment.

Human health is shaped not only by biological processes, but also by environmental and social conditions. Biomarkers including heart rate variability (HRV), cortisol, and blood pressure indicate current health and future disease risk within broader lifestyle and environmental contexts (7, 16) Nature exposure has been shown to positively influence these markers, offering strategies to improve multiple dimensions of health (Figure 1, panel 1) (13, 20). Nature-based care has formed basis for reinforcing theories such as the biophilia hypothesis, which posits an innate human affinity for natural environments (21). These insights have informed the incorporation of biophilic features into hospitals, schools, and workplaces, with documented benefits for stress reduction, learning, and productivity (21–27). Early implementation of nature as healing agents was seen in hospitals through therapy gardens, art installations, and water features. These natural sanctuaries led to measurable improvements in stress levels, happiness, and even recovery rates for patients (Figure 1, panel 2) (23–26, 28–30). From these successes, biophilic features have expanded across sectors. Schools use gardens to aid learning and academic engagement (27), while workplaces leverage natural light, and green views to boost productivity (22). This evidence propelled clinical conversations to include nature as medical prescriptions. In the 1980s, Japanese clinics introduced the concept of Shinrin-yoku encouraging immersive forest experiences to reduce stress and promote well-being (14). Formal GRx programs were introduced in New Zealand in the 1990s, followed by national and local initiatives in Oceania, the United States, Canada, and beyond (15, 31, 32). Despite growing evidence of health benefits, translation of nature-based interventions into equitable and scalable healthcare practice remains limited. Health systems face competing priorities and infrastructure constraints, while patients encounter barriers related to access, safety, and daily feasibility. Existing reviews focus primarily on clinical efficacy, often drawing from small or pilot programs, with less attention to implementation and healthcare integration.

The objective of this narrative review is to address this gap by synthesizing implementation-oriented evidence on GRx programs. Specifically, the review examines structural and environmental conditions shaping access to nature, healthcare system integration and clinical delivery mechanisms, and patient-level feasibility and participation. By identifying recurring implementation challenges and enabling factors, this review aims to inform pragmatic, practice-informed strategies to support more equitable and sustainable delivery of GRx.

## 3 Materials and methods

### 3.1 Review design and literature identification

This study employed a narrative review design to synthesize interdisciplinary evidence related to the implementation of GRx programs. The initial phase of the review sought to synthesize evidence of GRx across specific outcome domains, including cardiovascular health, obesity, and related chronic conditions. Early scoping of the literature revealed a growing body of research documenting associations between nature exposure and a range of physical and mental health outcomes. However, this preliminary evidence mapping also highlighted a notable gap. While the benefits of nature exposure were increasingly described, less attention had been paid to how GRx programs are implemented within and beyond healthcare systems.

As an emerging interdisciplinary field, the available evidence base was sporadic and diverse, spanning observational studies, qualitative research, program evaluations, policy documents, and conceptual frameworks. Absence of standardized interventions and outcome measures made a narrative review approach appropriate over a meta- analysis or a quantitative synthesis.

Accordingly, the scope of this review was refined to focus on implementation-oriented evidence related to GRx, with particular attention to structural, clinical, environmental, and patient-level factors shaping access, participation, and equity. These dimensions were considered collectively, as patient access, healthcare system integration, and follow-through are interdependent components of real-world GRx implementation rather than discrete processes.

A structured literature identification process was conducted to capture relevant evidence published over the past three decades. Peer-reviewed literature was identified through searches of PubMed, Scopus, and Google Scholar, selected to capture clinical, public health, and interdisciplinary research relevant to GRx implementation. To reflect the applied and practice-oriented nature of GRx programs, gray literature including government and institutional websites, policy documents, and implementation or evaluation summaries was also reviewed. This review did not involve the collection of primary data, human subjects, interviews, or original qualitative or quantitative analyses.

### 3.2 Search strategy and eligibility criteria

Search terms were developed iteratively and organized around three primary concept domains:

- Health outcomes (e.g., physical health, mental health, well-being, stress, cardiovascular disease, obesity, etc.,),
- Natural environments and exposure within urban environments (e.g., nature, forest, greenspace, parks, green infrastructure, biophilic environments) and
- Intervention and implementation constructs (e.g., green/nature prescriptions, nature-based interventions, access, adherence, clinical integration, urban planning, and public health systems).

These terms were searched independently and in Boolean combinations to identify literature spanning clinical medicine, public health, environmental health, urban planning, and design. Key publications were also manually screened to identify relevant sources not captured through database searches. To enable consistency within the search, explicit inclusion and exclusion criteria were defined as *a priori* and applied throughout the screening and synthesis process (Table 1). Iterative discussions were used to resolve uncertainties in eligibility.

**Table 1:** Inclusion and exclusion criteria used in literature search.

Inclusion criteria
• Focused on GRx or nature-based clinical interventions • Examined health outcomes related to nature exposure • Discussed clinical integration, frameworks, or policy • Peer-reviewed or gray literature • English language; published ≥1995
Exclusion criteria
• No human health or wellbeing outcomes • Non-transferable settings (e.g., wilderness-only contexts) • Non-empirical commentary • Unpublished materials • non-English publications

Eligible sources included empirical studies, qualitative research, observational analyses, program evaluations, policy reports, and conceptual or framework-based papers that addressed nature prescriptions or closely related interventions. Studies were excluded if they focused solely on theoretical discussions without application to health or healthcare contexts, or nature exposure without relevance to prescribed, structured, or programmatic interventions. Editorials, opinion pieces without substantive evidence, and studies lacking sufficient methodological or contextual detail were also excluded. These criteria were used to guide title and abstract screening, followed by full-text review where necessary, to ensure alignment with the objectives of this narrative synthesis.

For each included source, we extracted information on the study or program setting, geographic context, population characteristics, type of nature-based intervention or prescription, delivery or referral mechanisms, reported facilitators and barriers, and outcomes relevant to access, engagement, or implementation. Due to the nature of the review, and the scarcity of established programs, evidence was synthesized narratively rather than systematically, using a deductive, theme-informed approach to allow patterns and gaps to be more easily identified. In total, 22 papers were synthesized to identify key barriers to the implementation of GRx interventions within the broader healthcare system.

## 4 Findings

The narrative analysis revealed a persistent translational gap between clinician-initiated GRx and patients’ ability to meaningfully engage with these prescriptions in routine, real-world contexts. To organize these findings, the synthesis was structured around recurrent patterns identified across the literature, including:

- the structure and scope of GRx programs,
- multilevel barriers affecting translation from prescription to participation, and
- recurring implementation-relevant considerations that highlight where existing models succeed, strain, or fail in practice.

The pathways we used to synthesize our findings align with integrative biopsychosocial frameworks that conceptualize nature-health relationships as operating through interacting biological, psychological, and social mechanisms (33). Together, these themes inform the organization of the following sections and underpin the development of a practice-informed call-to-action framework, inductively derived from convergent patterns and gaps identified across the reviewed evidence.

### 4.1 Structure and scope of GRx programs

Across existing literature, GRx aims to connect clinical care with preventive public health strategies by recognizing greenspaces as a health-supporting resource. At their core, GRx consists of structured, evidence-informed recommendations from clinicians encouraging patients to engage in nature-based activities. These prescriptions range from generalized weekly goals to more specific tasks tailored to an individual’s abilities, preferences, and lifestyle. Some programs allow patients to select from curated activity options to support personalization and follow-through, while others incorporate structured follow-up elements such as check-ins or journaling to assess engagement and perceived impact (34).

GRx are consistently characterized as multifaceted, with program modules being applied to a range of issues including depression, anxiety, cardiometabolic disease, physical activity, along with others. Mental health practitioners increasingly incorporate nature-based activities to support stress reduction, mood regulation and management of neurodegenerative diseases (4, 5, 20, 35, 36). These approaches span passive integration such as conducting therapy sessions in shaded courtyards or garden settings to more active formats including walk-and-talk therapy (37). In parallel, GRx is frequently framed as a low-cost strategy to address rising levels of physical inactivity, a major contributor to hypertension, obesity, and cardiovascular disease. These examples are presented to illustrate longstanding cultural relationships between nature and health rather than as formal GRx models, highlighting the diversity of contexts in which nature-based care has been conceptualized outside clinical systems.

The literature also describes the use of GRxs in rehabilitation contexts. Green physical activity (GPA) prescriptions are reported to support physical recovery, including improvements in mobility and rehabilitation outcomes for musculoskeletal and neurological conditions (4, 38). Many GRx models are designed to extend beyond therapeutic settings and into patients’ daily lives, aligning with salutogenic care approaches that emphasize health promotion through routine environments rather than episodic clinical encounters.

**Figure 2:**
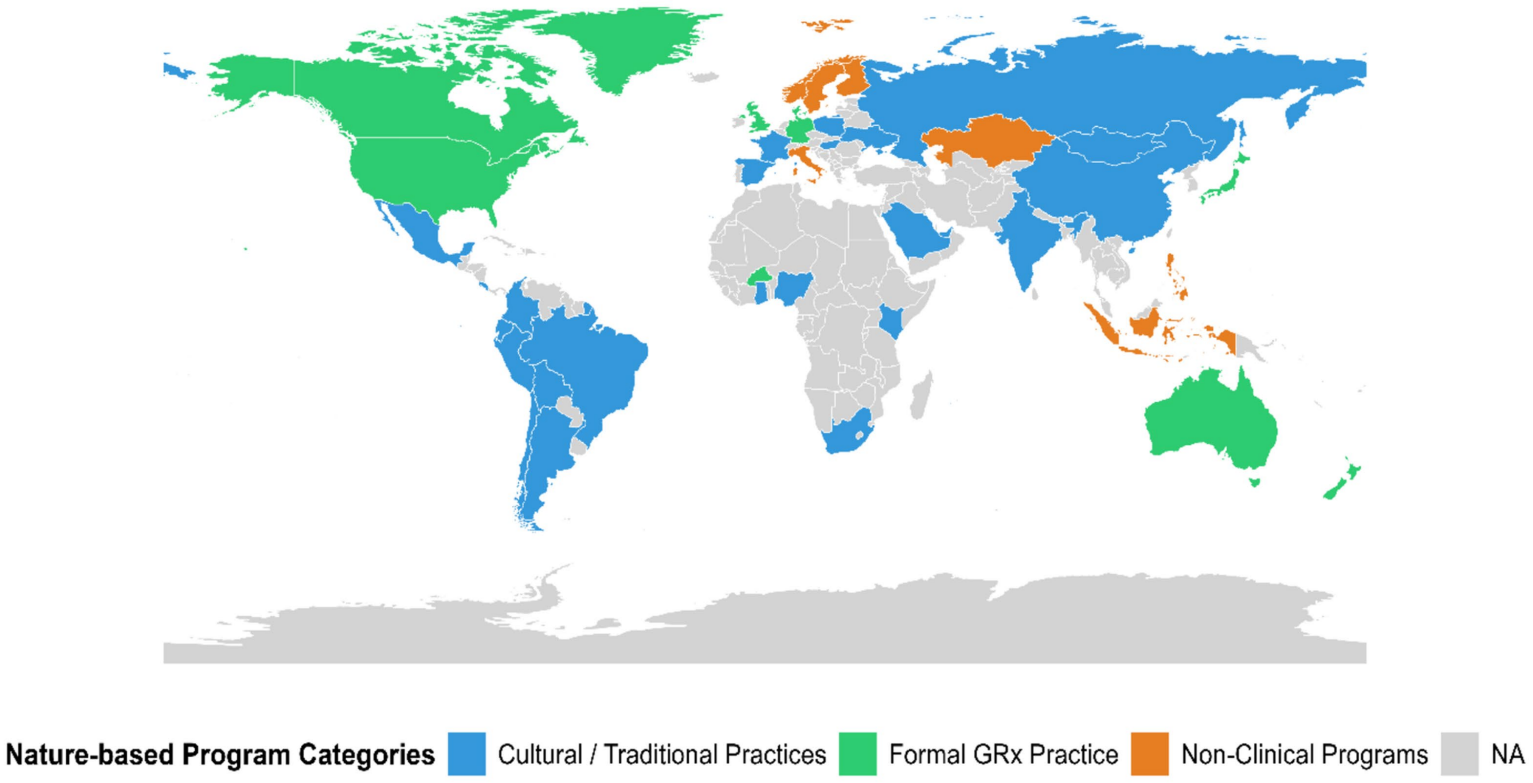
Illustrative global distribution of selected nature-based prescription (GRx) programs and practices identified through narrative literature screening. This map represents examples of GRx-related programs and practices identified during the narrative review process and is not intended to be an exhaustive or comprehensive inventory of all programs worldwide. Programs were categorized based on how they were described in source materials, rather than through systematic global program identification. Formal programs refer to initiatives funded, supported, or officially recognized by governmental agencies, healthcare systems, or national health organizations. Non-clinical programs include community- or nonprofit-led initiatives that promote nature engagement for health but are not formally integrated into healthcare delivery systems. Cultural or traditional practices refer to longstanding community-based, faith-based, or culturally embedded practices that incorporate nature or natural therapies for health and wellbeing outside of clinical contexts.

Globally, GRx has taken many forms. While some countries have formalized programs, others promote nature engagement through cultural or wellness practices. Formalized programs are most common in Oceania, Europe, and North America, where they are often supported by non-profit organizations, healthcare networks, or government-funded initiatives that link patients to outdoor activities either directly or through clinical referral pathways (Figure 2). In the United States, Park Rx America functions as an educational and implementation hub, offering clinician training resources and registries to support integration of nature prescriptions into care plans (39). In Canada, PaRx partners with organizations such as the BC Parks Foundation and the Canadian Medical Association to increase nature accessibility, with over 13,000 participating physicians offering patients free or discounted access to nature-based destinations (40, 41). New Zealand’s national GRx program, administered by the Ministry of Health, combines clinical referrals with peer coaching and community activity networks to support participation among adults and families (42).

In parts of the world without formal GRx systems, communities continue to engage with nature through traditional practices. In India, Ayurveda is a naturopathic practice that is founded on the harmony between human health and nature, contrasting western medicine (43). In communities across Africa, shamans use herbal remedies and rituals, embedded within the natural world in sacred forest groves (44). In Central America, Costa Rica embodies the way of “Pura Vida,” a lifestyle emphasizing frequent immersion in nature for rejuvenation and health (45).

Despite their success, these programs still serve a niche role within healthcare systems. While the diversity of GRxs highlights its adaptability across contexts, long-term effectiveness depends on whether patients can realistically engage with prescribed activities within their everyday environments. Even when endorsed by clinicians, many models rely heavily on individual capacity to navigate access, logistics, and sustained participation, setting the stage for the implementation challenges that hinder GRx from becoming a consistently equitable and effective healthcare tool.

### 4.2 Barriers to implementation

Literature points to a series of inter-related barriers to the implementation of GRx, spanning environmental conditions, structural access constraints, and clinical practice limitations. At the environmental and structural level, outdoor settings inherently differ from regulated clinical environments, which presents accessibility challenges for certain populations. Unlike clinical spaces, outdoor environments lack controlled conditions, limiting feasibility for individuals with autoimmune disorders, physical disabilities, or heightened vulnerability to environmental exposures. Climate-related factors have been shown to further compound these challenges. Extreme weather events including heatwaves, cold spells, and poor air quality can render outdoor spaces unsafe or impractical for extended periods of the year, particularly in regions already experiencing climate stressors (46). In addition, degradation of urban greenspaces including poor air quality, excessive noise, heat exposure, or competing uses such as tourism and events may reduce their therapeutic suitability and undermine the intended health benefits of nature-based prescriptions. Furthermore, communities facing economic disadvantage often lack proximity to safe, well-maintained, and usable greenspaces, limiting opportunities to act on prescribed activities (19). Transportation barriers, especially for individuals with mobility limitations, limited access to private vehicles, or inadequate public transit represent an additional constraint. For vulnerable populations, including children, older adults, immunocompromised individuals, LGBTQ+ and BIPOC communities, concerns related to physical safety, environmental hazards, supervision, harassment, and social exclusion may further restrict engagement in outdoor activities, even when greenspaces are physically accessible (31, 47). Even with safe access, patients may deprioritize GRx due to time and financial constraints. Incentive structures such as transportation support or insurance-based rewards may help offset participation barriers.

Barriers also emerge within clinical practice. Even in settings with viable green infrastructure, inconsistent physician engagement can limit GRx uptake. The literature describes limited clinician training, absence of standardized prescribing frameworks, and lack of integration into routine workflows as key constraints. In some cases, clinicians express support for nature-based care but hesitate to issue formal prescriptions due to uncertainty around liability or lack of institutional guidance, instead offering informal recommendations (19, 48). Acceptability related barriers are also reported including patient preferences for traditional pharmacological treatment or skepticism toward nature-based care, which may reduce engagement (49). Without system-level support, clinical normalization, or clear implementation pathways, GRx remains peripheral to mainstream care despite increasing awareness and interest.

### 4.3 Recurring implementation-relevant considerations for GRx models

Beyond identifying barriers, the literature reveals recurring implementation-relevant considerations that help explain why some GRx models demonstrate greater feasibility than others. These patterns highlight where existing approaches succeed, strain, or fail when translated into routine practice. A consistent theme concerns how responsibility for implementation is distributed. GRx models that rely primarily on individual patient initiative are frequently described as difficult to operate. These models unevenly distribute responsibilities by requiring patients to independently identify locations, manage logistics, and sustain engagement over time. In contrast, programs that extend responsibility through referral pathways, navigators, or community partnerships reduce logistical and cognitive burden on patients and are more often reported as feasible.

Integration within healthcare systems also shapes implementation. GRx models operating outside routine clinical infrastructure without documentation, referral mechanisms, or follow-up are commonly described as informal and episodic. Where GRx are embedded within clinical workflows, even in limited ways, the literature suggests greater alignment with provider practices and continuity of care (40, 46, 47). Community context further influences implementation. Programs that leverage local parks, nonprofit organizations, or culturally embedded practices are better positioned to address access, safety, and relevance simultaneously. However, these approaches are often localized, resource-dependent, and unevenly scaled, limiting broader generalizability. Finally, many GRx models also lack mechanisms for monitoring participation or adapting prescriptions over time. Without feedback or accountability structures, prescriptions risk becoming symbolic rather than actionable, contributing to inconsistent engagement and limiting evaluation of impact.

Collectively, these barriers indicate that GRx implementation challenges arise less from lack of perceived benefit than from misalignment between clinical intent and real-world feasibility. How support, infrastructure, and accountability are distributed across patients, clinicians, and systems therefore shapes whether GRx function as actionable care or remain aspirational. These patterns inform the practice-informed framework that follows.

## 5 Discussion: a call-to-action framework

**Figure 3:**
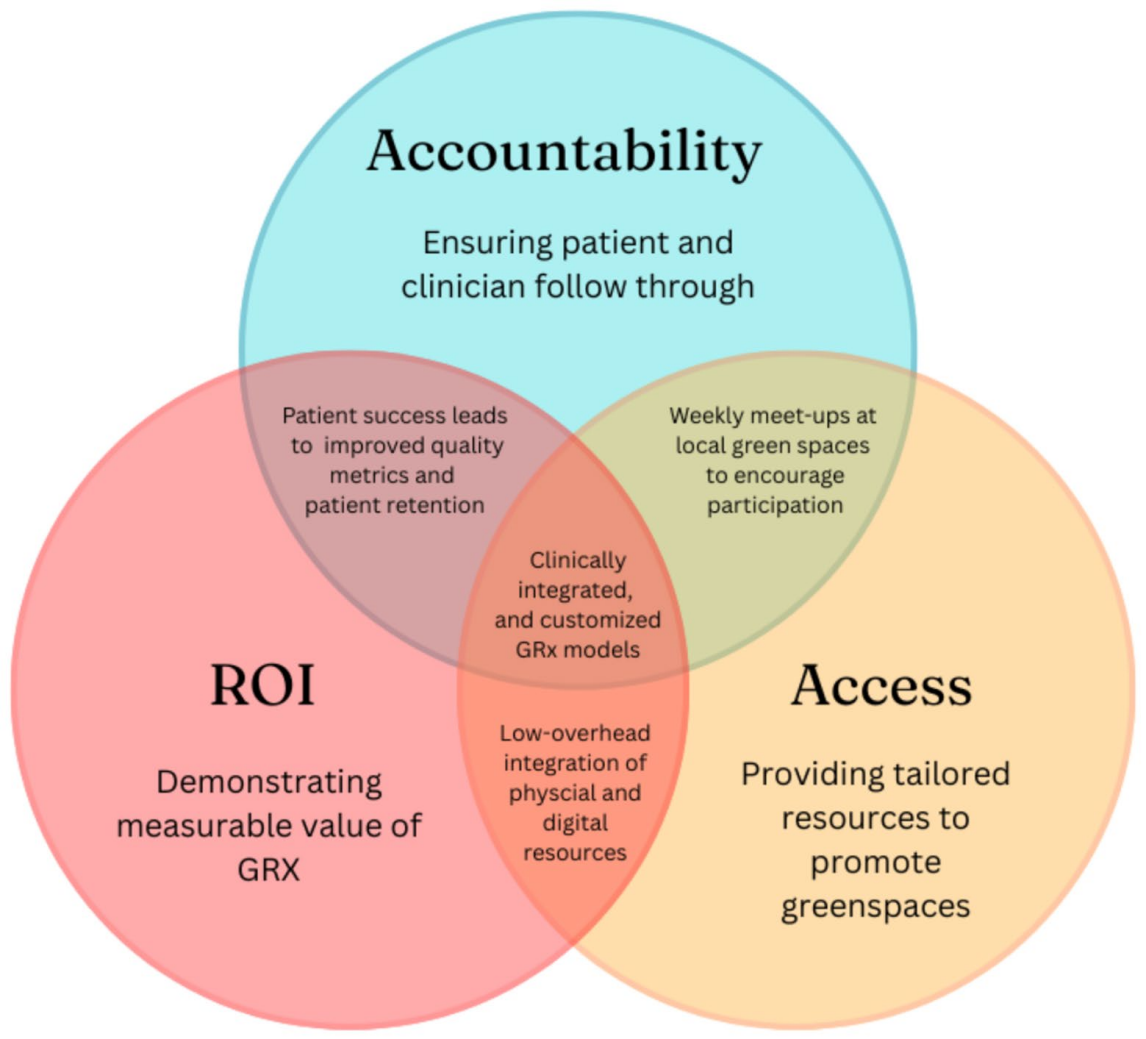
Practice-informed three-pillar framework for GRx implementation. The framework synthesizes recurring patterns identified across the narrative review and highlights three interdependent domains: access, value and return on investment (ROI), and accountability. These elements shape the feasibility of nature-based prescriptions in practice. The framework is intended as a conceptual guide to organize implementation considerations rather than a validated intervention model.

GRx cannot be effectively mainstreamed without addressing two persistent disconnects. One between clinical evidence and practice, and another between prescription and patient participation. Although GRx programs have expanded globally, the evidence synthesized here indicates that many patients remain unable to act on prescriptions due to structural, environmental, and system-level constraints. As urbanization, climate stressors, and health inequities increasingly shape access to nature, the feasibility of GRx depends less on clinical endorsement alone and more on how programs are operationalized within real-world healthcare and community contexts. In response, this call-to-action outlines a practical roadmap to support translation from concept to implementation through incremental, low-burden steps that clinics, hospitals, and community partners can adopt. The proposed framework (Table 2; Figure 3) is organized around three pillars as follows:

- Access to resources,
- Accountability
- Value and return on investment, and

**Table 2:** Proposed action framework to strengthen GRx access, integration, and accountability.

Challenge / gap	Recommendation / Action step	Stakeholders involved
Lack of awareness or training among clinicians (18, 31, 47, 48)	Provide continuing education, clinical frameworks, and plug-in tools for EHR integration	Hospitals, med schools, CE providers
Patients unaware of where, when, or how to engage with nature (18, 19, 40, 46)	Offer tailored resources based on ZIP code, mobility, and preferences	Clinics, health navigators, Park Rx orgs
Barriers due to safety, mobility, or transportation (19, 46, 49, 53)	Partner with local organizations to connect patients with accessible, culturally relevant, and safe spaces	Community orgs, YMCAs, parks departments
Underutilized local greenspaces (parks, trails, courtyards) (5, 6, 29)	Retrofit and enhance existing spaces using biophilic design principles	Urban planners, landscape architects, local gov
Inconsistent GRx follow-through and limited accountability (18, 46, 49)	Use patient portals, REDCap surveys, or text reminders to track engagement	Clinics, IT teams, public health staff
CHNAs overlook nature access (16, 53, 54)	Add canopy, walkability, and greenspace proximity indicators to CHNA frameworks	Hospitals, CHNA teams, GIS analysts
Greenspace development favors affluent areas	Reform zoning policies, mandate green % in new developments, and prioritize underserved areas	City planning departments, policymakers, grad students
Short-term projects lack long-term sustainability	Evaluate GRx infrastructure using Bardach & Patashnik’s criteria (impact, feasibility, maintenance)	Funders, policy analysts, health system leaders
GRx seen as niche or luxury intervention	Align GRx with Sustainable Development Goals (SDGs 3, 11.3, 11.7) and embed into public health infrastructure	Global health leaders, funders, NPOs

These pillars were inductively derived from recurring patterns in the narrative synthesis to guide scalable and context-sensitive integration rather than a one-size-fits-all model.

### 5.1 Access and feasibility

Access emerged as the most immediate and tangible constraint shaping whether nature-based prescriptions can be acted upon in practice. Prescriptions must connect patients to specific, nearby resources that align with their physical capacity, schedules, and neighborhood conditions. Several programs described in the literature emphasize the importance of moving beyond general encouragement toward place-based guidance that patients can realistically use. At a basic level, clinics and hospitals can start small by creating resource packets that map nearby parks, trails, arboretums, and community nature programs. These recommendations are scalable, offering general green space recommendations or personalized locations based on ZIP codes, such as local church trails or neighborhood gardens. These packets can include hours of operation, safety tips, walking difficulty, and transit options to ensure patient safety and adequate engagement. Digital resource programs like Park Rx America, local parks department directories, and third-party apps can be embedded into patient portals as supplementary resources.

Several models described in the literature could be propelled further through partnerships with wellness professionals or community navigators who can help patients identify nature-based options appropriate to their mobility, preferences, and daily routines. These partnerships allow prescriptions to be translated into individualized action plans without substantially altering clinical workload. In some settings, on-site or shared-use programs originally designed to reduce staff burnout have also been adapted for patient use, demonstrating how existing institutional resources can be leveraged with minimal overhead.

### 5.2 Build accountability and sustainability

The development of standardized GRx protocols that healthcare professionals can reference as clinical guidelines is essential to support consistency and clinical confidence. Currently, nature prescriptions rely on informal counseling or verbal encouragement, which risk being forgotten, ignored, or deprioritized within competing care demands. Embedding accountability mechanisms on both the clinical and patient sides of GRx delivery may improve adherence over time. Clinicians can begin by incorporating nature-based documentation into electronic health records (EHR), alongside referrals or medications. Adding intake questions like, “How much time do you spend outdoors each week?” or “Do you have access to a nearby greenspace? /Do you know where your nearest park is?” helps establish a baseline of patient interest and feasibility, while integrating nature-based language into routine workflows. When systematically captured, these data can inform GRx delivery, support clinical decision-making, and contribute to population-level planning. On the patient side, self-accountability plays a critical role. Self-monitoring tools such as checklists, journaling apps, or wearable devices can help patients track and log their GRx goals. Clinics may also deploy brief follow-up surveys through patient portals, using platforms such as REDCap or Qualtrics, to assess time spent outdoors, perceived benefits, and barriers. Some EHR systems allow patients to log nature-based activities directly within patient portals (50). To enhance engagement, health systems and insurers may consider modest incentive structures tied to participation, such as wellness credits, insurance premium reductions, or charitable donation matching linked to verified activity. Behavioral incentive models already used in step-count and workplace wellness programs may increase adherence while maintaining the preventive ethos of GRx (51). Together, these low-cost strategies support follow-through while minimizing additional administrative burden.

### 5.3 Health systems integration and value

To gain traction within mainstream clinical care, GRx must be framed not only as beneficial but also as cost-conscious, scalable, and patient-centered. Healthcare systems prioritize interventions with clear value propositions, those that reduce risk, lower costs, or improve quality metrics. Accordingly, the potential value of GRx as a complementary intervention, particularly within prevention-oriented and value-based care models, must be clearly articulated and supported by available evidence. Hospitals and clinics can support this shift by offering continuing education opportunities, recognizing participation through internal micro-credentials or departmental awards, and highlighting alignment with existing value-based care incentives. Platforms such as Park Rx America, Nature Sacred, and WELL v2 offer free, evidence-informed training modules that do not impose substantial time or budgetary burdens. Academic institutions can further support this transition by integrating nature-based health concepts into medical education and residency training. Over 90 universities, including institutions such as Cornell University, Texas A&M University, and UC Davis, participate in Campus NatureRx (CNRx) initiatives that prescribe nature through on-campus clinics (34, 52). These models illustrate how GRx concepts can be introduced with minimal curricular restructuring.

A limited but growing body of studies suggests that increased use of green spaces may be associated with reductions in healthcare expenditures in specific populations and contexts (17, 53, 54). For example, one study reported that the construction of an urban park was associated with over CAD 130,000 in reduced healthcare spending, attributed to increased physical activity, improved mental well-being, and reduced exposure to air pollution (55). In Australia, observational studies of office workers reported productivity gains of over ~11% following regular visits to national parks, with associated economic benefits at the population level, with an increase of ~1.8% of GDP, of which healthcare spending was reduced by 0.6% (56). These findings are context-specific and should be interpreted cautiously. Collectively, the evidence suggests that investments in accessible green infrastructure may yield broader public health and economic co-benefits.

Despite available evidence, there remains a need for more causal and longitudinal research to strengthen the scientific foundation linking nature exposure to specific health outcomes. Establishing stronger pathways may enhance clinical confidence, support policy adoption, and promote broader integration of GRx into routine care. For example, the ongoing Green Heart Project in Louisville, Kentucky is investigating how neighborhood greening interventions influence cardiovascular health outcomes, contributing to emerging causal evidence on environment–health relationships (57).

## 6 Strengths and limitations

This narrative review has several key strengths. First, it synthesizes evidence from diverse geographic contexts, drawing on studies and programs implemented across multiple regions globally. By incorporating examples from high-income settings as well as culturally embedded and community-driven practices, the review reflects the broad ways in which nature-based care is conceptualized and operationalized worldwide. Second, the review intentionally includes a wide spectrum of GRx models, ranging from formally structured, healthcare-integrated prescription programs to non-formal, community-based, and culturally rooted nature engagement practices. This inclusive approach captures how nature-based care functions both within and beyond clinical systems, acknowledging that many effective models operate outside standardized medical frameworks. Third, the review’s interdisciplinary scope integrating evidence from clinical medicine, public health, environmental design, urban planning, and community health allows for a more holistic understanding of implementation realities. This breadth is particularly important for GRx, where delivery and participation are shaped as much by environmental and social conditions as by clinical intent. Finally, the call-to-action advocates for incremental, low-burden, and context-sensitive actions that can be adopted across healthcare settings, rather than sweeping reforms or resource-intensive overhauls. This approach prioritizes practical steps that are adaptable across socioeconomic contexts, cultures, and levels of infrastructure, although recommendations would vary based on context. By focusing on feasibility and inclusivity, the review positions GRx as an intervention that can be strengthened without requiring overnight transformation of planning systems.

Like most studies, this paper also poses a few limitations. While the effects of nature on human health outcomes are well documented in scientific literature, the translation of this evidence in real-world healthcare implementation is limited. Accordingly, this review did not follow a systematic review or meta-analytic protocol, and no formal quality appraisal or risk-of-bias assessment was conducted. Consequently, included sources vary in methodological rigor, and findings should be interpreted as indicative rather than definitive. This approach also necessitated reliance on gray literature to capture real-world GRx implementation practices that are not well represented in peer-reviewed sources.

Second, the evidence base reviewed was heterogeneous, encompassing observational studies, qualitative research, pilot program evaluations, and conceptual frameworks with inconsistent definitions of interventions, outcomes, and implementation processes. Third, much of the available literature originates from high-income countries, particularly North America, Europe, and Oceania. While this may limit immediate generalizability, the experiences documented in these settings may also serve as early implementation models. Given that formal GRx programs remain limited in many regions, the lessons learned from high-income contexts can provide practical guidance for adaptation rather than a definitive blueprint. Finally, while the proposed call-to-action framework is informed by recurring patterns and gaps identified across the narrative synthesis, it has not been empirically tested or validated. The framework should therefore be viewed as a practice-informed synthesis intended to guide future research and implementation efforts rather than as an evidence-based intervention model. These limitations further highlight the need for more research, particularly large-scale, longitudinal, and implementation-focused studies, to examine how GRx can be implemented across diverse healthcare systems.

## 7 Future scope

While small-scale actions that have immediate impact are the core focus of this paper, future research and practice should examine the broader structural and systemic changes required to support GRx on a scale. Such work would help clarify how GRx can be sustained beyond individual programs and embedded within broader health and planning systems. While clinicians may be the ones writing GRx, our evidence suggests that the community plays a pivotal role in enabling implementation and long-term sustenance. To support widespread patient access, healthcare systems may benefit from partnering with local entities such as nonprofits, civic organizations, local governments, and urban planners already active at the intersection of health, equity, culture, and the environment. These partnerships can take many forms: faith-based organizations, libraries, schoolyards, and nature centers, often overlooked entry points of nature access. AgriLife programs across Texas, for example, offer guided walks, gardening clubs, outdoor fitness classes, or wellness programs (58). Future studies should evaluate how such partnerships influence participation, equity, and sustainability of GRx models.

Urban planning also plays a critical role in shaping access to nature. Zoning, land-use policies, and master plans influence where parks are located, how they are maintained, and whether features such as walking paths, shaded areas, or green buffers are prioritized. Future research should assess how participatory planning and community-engaged design approaches affect the usability and inclusivity of greenspaces, particularly in low-income and historically marginalized neighborhoods that continue to face disproportionate environmental burdens. One promising and cost-effective strategy involves retrofitting existing assets. Many communities already have parks, trails, schoolyards, or public lands that are underutilized or poorly maintained. However, empirical evidence is needed to determine how targeted, low-cost enhancements affect long-term use and health-related outcomes. Incorporating biophilic design features such as shaded seating, accessible trails, clear signage, water stations, denser foliage, and pet-friendly amenities may improve usability and perceived safety. Collaborations with Master Gardeners, landscape architecture students, or conservation nonprofits represent additional avenues for community-driven greening initiatives.

To scale nature-based care, greenspace may also need to be conceptualized and measured as a public health asset. Community Health Needs Assessments (CHNAs), required for nonprofit hospitals, offer a powerful but underutilized mechanism to assess environmental determinants such as access to nature. Traditionally, CHNAs emphasize clinical concerns such as diabetes, obesity, or mental health, but rarely examine environmental determinants like greenspace availability or infrastructure quality. Future work should explore standardized approaches for integrating indicators such as canopy coverage, park proximity, walkability, and frequency of engagement into CHNA processes. Existing toolkits, such as those developed by Practice Greenhealth provide a starting point, but further evaluation is needed to understand how these metrics inform planning, investments and equity-focused interventions.

Ultimately, reimagining nature as a public health tool opens multiple avenues for future inquiry. Embedding nature into clinical platforms may support more consistent prescribing, while partnerships with community organizations can reduce burden on clinical staff and support patient participation. Beyond direct health outcomes, future research should examine how GRx aligns with broader institutional priorities, including sustainability and social responsibility. In this context, positioning GRx within environmental, social, and governance (ESG) frameworks may facilitate cross-sector collaboration, though further empirical work is needed to assess how such alignments influence the long-term equity, scalability, and durability of GRx initiatives.
